# Co-Circulation of 4 Dengue Virus Serotypes among Travelers Entering China from Myanmar, 2017

**DOI:** 10.3201/eid2409.180252

**Published:** 2018-09

**Authors:** Binghui Wang, Yuebo Liang, Shuting Yang, Yirong Du, Li-Na Xiong, Ting Zhao, Fang Yang, Weihong Qin, Xueshan Xia

**Affiliations:** Kunming University of Science and Technology, Kunming, China (B. Wang, S. Yang, X. Xia);; Care Center for International Travel Health in Yunnan, Kunming (Y. Liang, W. Qin);; Ruili Entry-Exit Inspection and Quarantine Authority, Ruili, China (Y. Du, L.-N. Xiong, F. Yang);; First People’s Hospital of Yunnan Province, Kunming (T. Zhao)

**Keywords:** dengue virus, epidemic, cross-border travelers, genotype, serotype, viruses, Myanmar, Yunnan, China

## Abstract

We report 301 dengue virus infections among cross-border travelers entering Yunnan Province, China, from Myanmar during 2017. Phylogenetic analysis of 99 strains found all 4 serotypes co-circulating; genetic characteristics have also changed. This finding highlights the urgent need for monitoring dengue virus cross-border transmission as early warning of severe dengue fever.

Dengue virus (DENV) infection is one of the most serious threats to public health in tropical and subtropical regions worldwide (*1*). Southeast Asia is the most seriously affected region, with explosive outbreaks occurring frequently. In 2013, a small-scale DENV outbreak occurred in Yunnan, the southeasternmost province in China, as a result of imported infection from neighboring Southeast Asia countries (*2*,*3*). Ruili County is located in southwest Yunnan, bordering Myanmar on 3 sides. In this county, cross-border DENV transmission and endemic disease have become a serious challenge in the past decade.

In 2017, a total of 8.3 million cross-border travelers who entered Yunnan Province through Ruili had their body temperature measured using an intelligent infrared human body temperature measurement system. Persons with a temperature >37°C were suspected to be infected with DENV; infection was confirmed by NS1 antigen detection (*4*). In total, 1,667 travelers were screened for DENV infection because of fever symptoms. Of these, 301 were confirmed to be DENV infected. The DENV-infected travelers comprised 196 citizens of Myanmar and 105 citizens of China; median age was 27 (range 1–71) years, and male/female ratio was 1.15:1 (161:140). The occurrence of DENV infection was concentrated during August–November; 196 infections were detected in this period, accounting for 65.1% of DENV cases in 2017. 

To further describe the genetic characteristics of these DENV strains, we selected 100 DENV-positive plasma samples for E gene amplification followed by phylogenetic analysis (*2*). All participants were informed and provided written consent before sample collection. This research was approved by the Institutional Ethical Committee of Kunming University of Science and Technology. We randomly enrolled 10 cases from each month; if there were <10 cases in 1 month, we included all cases from that month. As a result, 99 samples were successfully amplified; 1 sample failed, possibly because of low viral load. We identified all 4 DENV serotypes in this population, although DENV-3 (4 cases) and DENV-4 (17 cases) had previously been undiscovered in Yunnan Province (*2*). DENV-1, the dominant serotype in 2013, continued to be the most prevalent serotype in 2017 (77 cases), whereas only 1 case of DENV-2 was detected. We found no significant difference in serotype distribution based on sampling time.

We randomly selected 40 DENV-1 samples, 1 DENV-2 sample, 4 DENV-3 samples, and 17 DENV-4 samples for sequencing and phylogenetic analysis; we submitted the resulting sequences to GenBank (accession nos. MG933806–MG933867). Phylogenetically, all DENV-1 strains of the cross-border travelers in 2017 were classified as genotype I (Figure), similar to earlier reports (*2*). Specifically, most strains were closely related to the strains identified in 2013 in Dehong Prefecture and its neighboring country, Thailand (Figure), which suggests the prolonged circulation of 2013 strains or stable importing from neighboring countries. The only DENV-2 strain (D2-0022) was classified as the Asian I genotype, forming a close cluster with 2013 strains and reference sequences from Myanmar and Thailand and strains previously circulated in Yunnan Province. The DENV-3 strains detected in Ruili were classified as genotype I and genotype III, which is substantially different from the genotype II strains identified during the DENV outbreak in the southern prefecture of Xishuangbanna in 2013 (Figure) (*2*). DENV-4 genotype II was once reported as a circulating serotype in Yunnan Province in 2015 but is now a long-term epidemic in Myanmar and Thailand.

**Figure:**
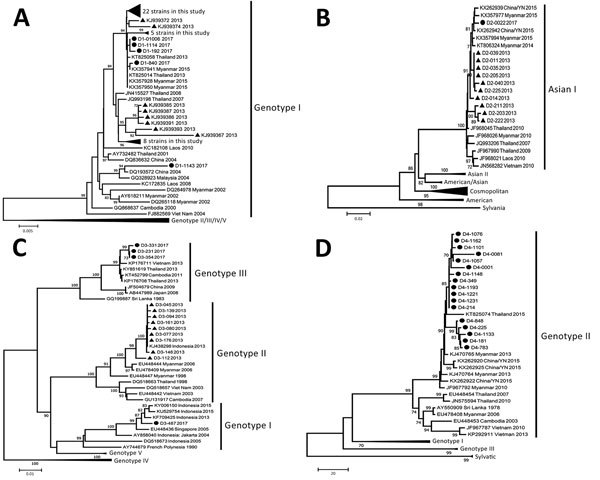
Phylogenetic tree of DENV serotypes identified in cross-border travelers entering Yunnan Province, China, from Myanmar during 2017. A) DENV-1; B) DENV-2; C) DENV-3; D) DENV-4. The phylogenetic trees were constructed by the maximum-likelihood method with a Kimura 2 parameter model using MEGA 7.0 software (https://www.megasoftware.net*).* Bootstrap values were set for 1,000 repetitions. Black dots denote strains from this study, and black triangles denote strains from our previous study (*2**,**3*). GenBank accession numbers for comparison isolates are provided. Scale bars indicate nucleotide substitutions per site. DENV, dengue virus.

Among the travelers entering Yunnan Province from Myanmar in 2017, dengue infections showed not only inherited characteristics of previous epidemic DENV-1 and DENV-2 but also the circulation of additional serotypes and genotypes (DENV-3 genotypes I and III, DENV-4 genotype II). This importation of all serotypes of DENV may result in simultaneous or sequential epidemics of the local population in Yunnan Province. Co-circulation of the 4 serotypes, considered a key indicator of progression toward hyperendemic transmission (*5*,*6*), led to an alert for a threatening DENV pandemic. Our findings also revealed the continued changing of DENV genetic characteristics in this float population (*5*,*7*,*8*), developed from fewer genotypes/serotypes to the co-circulation of multifarious serotypes/genotypes, from sporadic imported infection to more local infection. In the past decade, or at least since 2013, local DENV infection has occurred in border regions of Yunnan Province. Thus, DENV undoubtedly also exists in local mosquitoes. In the near future, we plan to investigate DENV infection in mosquitoes and perform genetic characterization of those strains from mosquitoes.

The cross-border population serves as a major vector for transmission of pathogens into China from neighboring countries in Southeast Asia. Because of the large number of DENV infection cases in Yunnan Province and neighboring countries, our research clearly demonstrates that surveillance and control of dengue virus is a difficult task, and south China is under the risk for an increase in dengue infections.
